# Human Subjects Protection and Technology in Prevention Science: Selected Opportunities and Challenges

**DOI:** 10.1007/s11121-016-0664-1

**Published:** 2016-05-24

**Authors:** Anthony R. Pisani, Peter A. Wyman, David C. Mohr, Tatiana Perrino, Carlos Gallo, Juan Villamar, Kimberly Kendziora, George W. Howe, Zili Sloboda, C. Hendricks Brown

**Affiliations:** University of Rochester, Rochester, NY USA; Northwestern University, Chicago, IL USA; University of Miami, Coral Gables, FL USA; American Institutes for Research, Washington, DC USA; George Washington University, Washington, DC USA; Applied Prevention Science International, Inc., Ontario, OH USA

**Keywords:** Human subjects, Prevention, Technology, Ethics, Common rule regulations, IRB

## Abstract

Internet-connected devices are changing the way people live, work, and relate to one another. For prevention scientists, technological advances create opportunities to promote the welfare of human subjects and society. The challenge is to obtain the benefits while minimizing risks. In this article, we use the guiding principles for ethical human subjects research and proposed changes to the Common Rule regulations, as a basis for discussing selected opportunities and challenges that new technologies present for prevention science. The benefits of conducting research with new populations, and at new levels of integration into participants’ daily lives, are presented along with five challenges along with technological and other solutions to strengthen the protections that we provide: (1) achieving adequate informed consent with procedures that are acceptable to participants in a digital age; (2) balancing opportunities for rapid development and broad reach, with gaining adequate understanding of population needs; (3) integrating data collection and intervention into participants’ lives while minimizing intrusiveness and fatigue; (4) setting appropriate expectations for responding to safety and suicide concerns; and (5) safeguarding newly available streams of sensitive data. Our goal is to promote collaboration between prevention scientists, institutional review boards, and community members to safely and ethically harness advancing technologies to strengthen impact of prevention science.

Advances in Internet-enabled connectivity and computing offer new opportunities (Brown et al. 2013; Mohr et al. 2014) across all phases of the prevention research cycle (Kellam et al. 1999), from generative research to the dissemination and implementation of interventions. Technological advances also create new opportunities to promote the welfare of human subjects. Proposed changes to modernize the federal rules that govern human subjects research in the USA (US Department of Health and Human Services 2015) underscore the need to re-evaluate and update our operating procedures (Behnke 2006) in prevention science to encourage broad and well-informed research participation in a digital age.

In this article, we begin by briefly reviewing key guiding principles pertaining to prevention research with human subjects and proposed changes to the rules that govern such research in the United States. After establishing this context, we focus on benefits afforded by technology to promote these principles through the following: (1) research with hard-to-reach populations through new venues and delivery platforms and (2) data collection and interventions that reach more broadly and deeply into participants’ lives. We then outline five human subjects challenges that prevention researchers must surmount to realize the gains that technology-enabled research promises, and offer technological and other solutions to these challenges that can empower participants and strengthen the protections that we provide. The challenges are as follows: (1) achieving adequate informed consent with procedures that are acceptable to participants in a digital age; (2) balancing opportunities for rapid development and broad reach, with gaining adequate understanding of population needs; (3) integrating data collection and intervention into participants’ lives while minimizing intrusiveness and fatigue; (4) setting appropriate expectations for responding to safety and suicide concerns; and (5) safeguarding newly available streams of sensitive data. Our goals are to help prevention scientists harness advancing technologies to strengthen human subjects protections and expand the impact of prevention science.

## Salient Human Subjects Challenges in Prevention Science

The Belmont Report (US Department of Health Human Services 1979) was a response to ethical malfeasance. These principles now govern human subjects research across federal agencies that fund or conduct research (US Department of Health and Human Services 1991) and serve as a guide for integrating Internet-based technology into prevention research practices. Below, we briefly review these principles as they pertain to prevention research and discuss current efforts by the US government to modernize the federal rules that codify these principles.

*Justice* emphasizes equitable selection of participants, so that both the burdens and benefits of research are fairly distributed. In prevention research, concerns about justice arise in several ways. For example, research has historically under-represented women and minorities (Yancey et al. 2006). The term “scientific equity” describes the need for equality and fairness in the scientific knowledge produced which can lead to empirically driven policies to overcome disparities (Brown et al. 2013; Perrino et al. 2014). One threat to justice in prevention science is the risk of inadvertently increasing stigma against a target subpopulation, as happened when research on alcohol use among the Inupiat Indians of Barrow, Alaska (Foulks 1989), was leaked and sensationalized, causing economic and other harms (Hodge 2012). This problem is revisited with new communication technologies because they simultaneously provide avenues for promoting justice by reaching underserved and marginalized populations, such as lesbian, gay, bisexual, and transgender (LGBT) youth (Silenzio et al. 2009), while unearthing controversial, possibly stigmatizing data.

*Respect for persons* centers on research participants’ autonomy, as well as the researcher’s obligation to protect those with diminished autonomy (e.g., children or disabled individuals). Meaningful interaction is at the heart of an informed consent process that achieves respect for persons. Informing participants about the requirements and risks of participation is necessary, but assuring “understood consent” (Bhutta 2004) is the true goal. The researcher bears responsibility for determining a candidate’s competence, comprehension, and appropriateness for the study.

The population health focus of prevention research can present special challenges for protecting autonomy and ensuring informed consent. Sometimes, formal consent of every individual affected by a large-scale intervention is unachievable, as with group-based preventive interventions that target entire communities (Wyman et al. 2014). As discussed below, respect for persons can be advanced by new technology-enabled methods for disseminating study information, ensuring comprehension, and monitoring ongoing consent and opt-out decisions. At the same time, technology use raises ethical dilemmas when it allows researchers access to information about participants’ lives with minimal interaction, as when interventions are embedded deeply and invisibly into daily routines.

*Beneficence* emphasizes the obligation to maximize possible benefits and minimize possible harms to individuals and society, including a loss of knowledge if the research is not undertaken at all. A key aspect of beneficence is the mandate to “do no harm.” Prevention researchers face particular challenges here because their activities frequently provide intervention to large populations in which most are asymptomatic and will not become disordered. A leading prevention science paradigm focuses on reducing malleable risk processes antecedent to disorders (Kellam et al. 1999) in broad populations (i.e., universal prevention). Evidence that some universal interventions can be beneficial to certain population subgroups while harmful to others (e.g., universal middle school substance abuse prevention; Sloboda et al. 2009) underscores the tension between adherence to the principle of beneficence and the need to seek and understand strong conceptual theories. The problem is not new. Similar challenges have appeared in programs targeting adolescents at risk for eating disorders by exposing low-risk youth to information which may undermine healthy eating habits (e.g., learning that purging is used by some in an attempt to manage weight, O’Dea and Abraham 2000). As discussed below, the ability to monitor progress remotely and respond to safety concerns promotes beneficence in prevention research. Conversely, conducting research with participants remotely introduce risks of a mismatch with population needs and, therefore, a potential risk of iatrogenesis.

*Respect for privacy and confidentiality* are core to human subjects protection and cut across all three of the Belmont principles. Privacy is the right to control access to information about oneself. As a personal right, it is defined subjectively, making standards fluid and culture bound. The regulations governing privacy are based on “reasonable expectations” that participants are not being observed or recorded or that information that they provide will not be made public (US Department of Health and Human Services 1991). In prevention research, privacy is most often threatened in the recruitment process, when potential participants may be approached based on information or in contexts that they do not wish to be known, such as a health clinic or juvenile court records, or information posted to a social media website.

Confidentiality refers to the protection of identifiable information. As discussed below, advances in technology make it possible to protect certain aspects of participants’ privacy and confidentiality more effectively than ever, while paradoxically putting other personal information at greater risk of disclosure. Modern security measures can guard against accidental disclosure and data theft, but large streams of data mean greater risk of participants being identified based on combinations of reported or released information, known as deductive disclosure (Sieber 2006).

### Federal Policy for the Protection of Human Subjects: NPRM

Although the core principles of human subjects protection remain relevant and binding over time, the application of these principles must evolve with the state of science, society, and technology. In September 2015, sixteen federal agencies issued a Notice of Proposed Rulemaking (NPRM, US Department of Health and Human Services 2015) with the aim of modernizing, strengthening, and streamlining the federal policy, known as the Common Rule Regulations (US Department of Health and Human Services 1991). The NRPM, along with an executive summary of the goals and provisions, is available on the HHS website (US Department of Health and Human Services 2015).

While extensive policy-making steps lie ahead and ramifications are not yet known, the Notice of Proposed Rulemaking (NPRM) signals the direction in which human subjects regulation is going (e.g., Emanuel 2015; Hudson and Collins 2015) and therefore merits discussion with regard to technology in prevention science. Three broad areas are relevant. First, NPRM proposes to make informed consent documents more transparent and concise and would require researchers to post consent documents on a public government website. New standards present an opportunity for updating online consent processes, which heretofore have mostly mimicked the length and density of traditional paper documentation. Computer and phone screens provide a blank canvas for attractive and interactive audio or visual consent presentations, potentially aiding transparency and communication. But, as discussed below, challenges remain for figuring out how to keep participant burden low and interactions brief. Second, a number of proposed changes could reduce institutional review board (IRB) oversight of lower-risk online interventions, potentially increasing the degree to which prevention researchers will be trusted to self-monitor. For example, NPRM creates new categories of excluded research, including “benign interventions with adults” and “secondary use of identifiable private information that was collected for non-research purposes.” The NRPM would also reduce IRB oversight for other low-risk research, including the proposal to eliminate continuing review for many studies. Third, for low-risk studies where confidentiality is the primary concern, the NPRM would decrease the IRB role and shift the burden of participant protection to data security teams—allowing IRBs to focus their attention on higher-risk studies. Changes along these lines could increase interaction between prevention researchers and data security experts and enhance the need for researchers to have the requisite background knowledge to evaluate options offered by technical experts.

## Opportunities: New Venues, Delivery Platforms, and Populations

Researchers now have access to populations of individuals around the world via Internet-networked communication devices. Delivering effective prevention programs to minority, marginalized, and geographically remote populations holds enormous potential for reducing health disparities and promoting justice and scientific equity in human subjects research (Brown et al. 2013; Muñoz 2010; Perrino et al. 2013). By the end of 2014, 40 % of the world’s population will have wired-broadband Internet access (International Telecommunications Union 2014). Mobile access is increasing even faster and is expected to reach 2.3 billion globally within the next year (International Telecommunications Union 2014). Adoption of mobile Internet-connected devices among US minority groups is especially rapid. Although legitimate concerns exist that some subgroups could get left behind, minority and marginalized groups that might have missed benefits from previous technologies appear to be participating robustly in the mobile revolution. A greater proportion of African Americans and Latinos than Whites use their mobile devices as their primary means of accessing social networking, email, and entertainment. The gaps in overall Internet access between Whites and minorities (Smith 2015) and young and elderly (Gilleard et al. 2015; Smith 2014) are disappearing, and other marginalized groups such as immigrants, migrant farm workers, and homeless youth are active users of mobile technology (Price et al. 2013; Rice et al. 2011; Welcoming Center for New Pennsylvanians 2012). Broader participation in prevention research benefits society, since scientific knowledge will be more widely generalizable.

Online recruitment and intervention occur primarily via three online platforms, each of which has distinct advantages for reducing disparities: public websites and services, online software retailers, and social media. A growing number of studies have demonstrated the efficacy of delivering interventions on these platforms (Mohr et al. 2013a). First, self-help websites can attract populations seeking interventions. For example, Muñoz and colleagues reached individuals across the English- and Spanish-speaking world with a public self-help website that has proven successful in reducing smoking (Muñoz et al. 2006). The website was free and open to the public and invited voluntary “opt-in” participation in research on the program. Websites dedicated to particular health issues provide opportunities to identify and recruit at-risk individuals amenable to online interventions. For example, Mood Gym (Christensen et al. 2004) teaches cognitive behavioral skills to prevent depression, providing opportunities to identify and recruit at-risk individuals amenable to online interventions. Crisis text services, such as Crisis Text Line (Crisis Text Line 2015) and the Veterans Crisis Line (US Department of Veterans Affairs 2014), attract new populations of at-risk individuals and generate vast quantities of data researchers that can use to understand the needs of individuals in crisis and discover new ways to help them in the short and long term.

Second, online retail sites, such as the Apple App Store, give access to large, active, customer bases. These are new venues for research and intervention delivery. A growing number of researchers are releasing their research applications on these stores. Some require potential participants to contact a research coordinator to unlock the app, while others invite people to participate in the study but allow those who do not want to consent to continue to use the apps. Apple released ResearchKit, an open-source software framework that supports in-phone consenting and manages assessments (Ritter 2015), followed by announcements from companies that will port the platform for use with the Android smartphone operating system (e.g., Patel 2015).

The format for software-based interventions can vary widely—from highly text-based versions of existing health-promotion programs (e.g., a virtual behavior therapy coach (Rizvi et al. 2011) to graphics-based video games (e.g., a diabetes management game for children that involves running from and chasing monsters (Garde et al. 2015). The nexus of prevention and commerce has created new opportunities for partnerships between academic researchers and commercial entities, bringing resources to accelerate the development of research-supported and evaluated interventions (Mohr et al. 2013a), reducing disparities (justice), and increasing the public health impact (beneficence).

Third, social media applications (Facebook, Twitter, Instagram, Qzone, and Weibo) have fundamentally changed how people connect socially, creating new virtual communities. These communities offer access to difficult-to-reach populations and the opportunity to study network effects, which have demonstrated importance in prevention (Valente 2010). For example, suicide prevention researchers used network mapping over MySpace to identify and contact a “hidden population” of LGBT youth at risk for suicide (Silenzio et al. 2009). Network recruitment methods include “respondent-driven sampling” (Homan et al. 2013) to identify and recruit hard-to-reach, at-risk populations. Similarly, sexual health researchers have used Grindr, a messaging application geared toward gay and bisexual men, to target and recruit men who have sex with men (Burrell et al. 2012; Landovitz et al. 2013; Rice et al. 2012) Remote recruiting avoids the stigma that some participants experience with in-person recruitment, thereby reducing potential harm and burden (beneficence). In addition to these existing platforms, the National Institute of Health announced new funding for mobile health research infrastructure (National Institutes of Health 2015). This infrastructure, along with NPRM rules that would exclude several new categories of low-risk research from IRB review, is likely to accelerate mobile health research and ensure that mobile-mediated participant recruitment, consent, and intervention will become increasingly broad and common.

## Opportunities: Interventions and Data Collection: Anywhere and “Everyware”

Networked communication devices—computers, smartphones, and sensing devices (e.g., geolocation or biosensors) that send and receive information over the Internet—open up new possibilities for promoting the welfare of human subjects. Everyware (Greenfield 2006) refers to a state of society and technology (rather than any particular class of hardware) in which networked devices become so ubiquitously embedded into everyday objects that information processing “dissolves in behavior.” Currently a theoretical extreme, Everyware identifies an ongoing trend. Passive sensing and data collection capability is already in many everyday objects—thermostats that detect movement and living patterns, light bulbs that change color when your spouse pulls into the driveway, and watches that track heart rate and activity. This deep integration of software provides ever-increasing ways to both collect and distribute prevention information. First-generation research is underway, for example, to use phone-based activity sensor (accelerometer) data to detect and respond to depression cues, such as decreased movement/activity (Saeb et al. in press), and contact lenses to continuously monitor blood sugar levels (Otis and Parviz 2014). For the intervention opportunities to be realized, an enormous amount of newly available, individualized data will have to be mined, interpreted, and responded to, but the technological capability is there.

Prevention research that leverages a broad array of Everyware devices will have distinct advantages for monitoring and responding to concerns about safety, iatrogenesis, and implementation quality, while simultaneously reducing participant burden. First, networked devices can give investigators access to an ongoing stream of information about participant risk, safety, and responses to intervention—generating early decision points. Such “streaming” could allow researchers to detect adverse responses or safety risks more quickly, rather than learning of poor responses only after endpoint data are collected and analyzed. In the case of safety or suicide risks, networked devices can be used to communicate key data, such as location, identity, and symptoms in emergency situations. Natural language-processing researchers have begun testing software to detect suicide concerns in text-based communication and to alert proctors immediately (Dinakar et al. 2015; Pestian et al. 2010). Other safety concerns, such as online bullying, can be detected in a similar fashion (Dinakar et al. 2012). Practical challenges remain; however, it is at least theoretically possible for researchers to use passively collected streams of data to mitigate risks, adjusting or initiating additional communication while a trial is still in progress. In most of our current non-technologic preventive interventions, we generally make only one type of error: not recognizing risk when it is there. Technology-based monitoring may potentially reduce this failure-to-recognize type of error but could increase false positives and result in other types of harm, including stigma.

In a similar vein, technologies can help researchers promote implementation quality, even for programs delivered in local communities. Implementation scientists are developing methods that use smartphone microphones, voice recognition, and computational linguistics to study and enhance the fidelity and competence with which community implementers deliver intervention components. Transcripts of family visits are scanned automatically for linguistic patterns that are linked to high fidelity (Gallo et al. 2015). Further developing such capabilities is critical because the welfare of participants depends on the quality and safety of prevention program delivery.

Finally, specific tailoring and “unobtrusive measures” (Webb 2000) have the potential to reduce participant fatigue and burden—key aspects of beneficence. Even universal interventions can be personalized because access is at an individual level. Tailoring can reduce time wasted on irrelevant or mismatched material. For example, we anticipate that technology-supported tailoring will allow scaling of highly effective parenting programs (e.g., Pantin et al. 2009; Wolchik et al. 2013). These programs have been challenging to deliver to large portions of the population because they require specific matching to family needs. Participant burden can also be reduced with computerized adaptive testing, which greatly diminishes the number of items necessary to achieve reliability (Gibbons et al. 2008), as well as passive data collection. Ambient, wearable, or implantable devices allow participants to “set it and forget it.” Access to multiple streams of real-time data has the potential to deliver interventions at the most impactful times and places, only requiring participants’ time and attention in a targeted fashion (Pejovic and Musolesi 2014). Such use of real-time data to inform real-time delivery of an intervention is known as just-in-time, adaptive interventions (JITAIs, Nahum-Shani et al. 2014). For example, we envision just-in-time intervention to prevent the spread of HIV among intravenous drug users using geolocation sensors (Brown et al. 2013).

### Challenge No. 1: Achieving Adequate Informed Consent with Procedures that Are Acceptable to Participants in a Digital Age

Changes to commonly used informed consent practices are needed to achieve the benefits mentioned above and the transparency and reduced burden that NPRM aims for (see above). Traditionally, participants are presented a single detailed document containing a study’s purpose, risks, benefits, data storage, confidentiality, and compensation plans. Comprehensive consent is requested at first contact with the participant.

Although providing highly detailed information upfront theoretically promotes autonomy, applying this approach in the digital setting often clashes with user expectations. This can result in reduced participation and biased samples, which reduce the scientific value and waste participant resources—a failure to promote beneficence. For example, about half of participants who downloaded a mood management app from the Google Play store (Center for Behavioral Intervention Technologies 2015) as part of a quality improvement project refused to sign an in-app consent by typing in their name, to allow researchers to collect usage data (D. Mohr, personal communication, May 18, 2015). Opt-in research participation involving publicly available websites and apps is increasingly common, making it a good target for standardization, with procedures that are acceptable to participants in a digital age.

Second, presenting consent information in a comprehensive manner at first contact can result in diminished comprehension. The most common strategy for achieving consent in online studies is to present an extensive “click-through” agreement (an information screen with a button to signal agreement), but these can be problematic. Research has demonstrated that users are disinclined to read long-form text on a computer screen and tend to misjudge their comprehension compared to the same text printed out (Ackerman and Goldsmith 2011). Instead, people reading electronic content on smaller devices employ a “scan and skip” approach. Less than 50 % of adults presented with a typical click-through actually read the entire document before clicking to continue (Böhme and Köpsell 2010).

Researchers at Facebook, Inc. and Cornell University investigated the network spread of positivity and negativity by manipulating some of their users “News Feeds” (running list of posts by friends) and measuring the valance of users’ subsequent posts (Kramer et al. 2014). Even though the study “was consistent with Facebook’s Data Use Policy to which all users agree” (Kramer et al. 2014, p. 8789), the study still caused controversy. There was widespread criticism in the media, scrutiny from congress and the FCC, and an eventual expression of concern from the journal’s publisher. The Cornell University IRB did not review the study because faculty had access to results, but not individual data (Cornell University Media Relations Office 2014). The lead researcher later acknowledged, “I can understand why some people have concerns about it, and my coauthors and I are very sorry for the way the paper described the research and any anxiety it caused. In hindsight, the research benefits of the paper may not have justified all of this anxiety.” (Kramer 2014)

Highly publicized episodes like this one undermine public confidence, and the NPRM seeks to increase transparency to avoid such problems. In the case of Facebook, the click-through for research participation was very general and hidden in the initial user registration process. Click-through agreements may be appropriate when the potential risks of participation are minimal or when more elaborate consent procedures would be burdensome, off-putting, or unfeasable. But, even in these cases, researchers can take steps to improve comprehension and decrease the chance of error or manipulation (Kunz et al. 2001). Best practices include the following: allowing participants to view informed consent information in digestible chunks and in easy-to-understand ways, giving participants the ability to review terms after starting participation, offering participants a choice between assent and rejection (not just, “click here to accept” but offering a “do not accept” button), labeling buttons in meaningful ways (“yes” or “I agree” rather than “continue,” “submit,” or “enter”), and providing participants with notice of the consequences of assent or rejection.

#### “Critical Junctures” Approach

One alternative to both simple click-through agreements and overly burdensome initial consent procedures is what we call a *critical junctures* approach. Consent need not be a comprehensive, one-time occurrence. Although a comprehensive initial consenting process is appropriate for some studies (e.g., a trial of a new networked glucose monitor or surgical procedure), many studies could protect participants better by providing information and soliciting consent as the participant reaches critical junctures. Critical junctures, identified for each study a priori as part of human subjects protocols, could include the start of data collection, the start of an intervention module, the first time that a certain type of data are stored (e.g., GPS data), or at the close of the study. In this way, consent information is integrated with other communications taking place within the natural flow of an intervention. This approach is consistent with current practices on many mobile devices. For example, on both Android and Apple iOS devices, users give permission for an app to use a microphone, camera, or GPS sensor just prior to the first use by an application—not in one long consent screen the first time that the app is launched. In the case of Facebook, the company could include a broad consent to research in their terms of service agreement that allows users to select types of research that they would like to opt into, then present a simple dialog box when they wish to conduct a new study or use data collected in the ordinary course of business for scientific purposes.

For studies with experimental conditions or greater intrusiveness, key elements of concern can be highlighted in pictorial or video form, at critical junctures. Long-form consent documents can be provided on request, while still presenting online information in brief bullets, infographics, or videos. These changes require researchers and IRBs to define and agree on elements needing special attention and those that may be omitted. Because the cost of making and revising mixed-media consent materials is higher than text, an iterative process incorporating IRB review may be required. Evolving electronic tools offer another approach to consent. For example, EduConsent™ (Systemedicus Inc. 2015) is an iPad-based system using continuous video documentation of a consent session: participants view videos and respond to questions at significant steps to demonstrate understanding. Although not yet widely available, adoption of such tools could markedly reduce variation across researchers and increase true informed consent.

### Challenge No. 2: Balancing Opportunities for Rapid Development and Broad Reach with Gaining Adequate Understanding of Population Needs

Greater access necessarily implies the potential for greater harm when an intervention does not match the needs of a population. Thus, matching tech-enabled prevention programs to population needs and culture is critical. Unfortunately, remote interaction can distance researchers from the communities that they hope to serve. Geographical and social context may be missing, leading to cultural mismatches, misunderstandings, and conflicts with family or community values. Fewer direct contacts with research participants in their natural environment also mean fewer natural mechanisms for detecting and correcting these problems. High dropout rates in online interventions (Muñoz et al. 2006) are difficult to interpret when participants are not being observed directly. Lack of follow-through might simply indicate “window shopping” behavior, similar to examining but not purchasing a self-help book in a bookstore, but there is a potential for unaddressed harms.

Limiting interactions to computer-mediated only has the potential to erode the human connection, empathy, and engagement between researchers and participants, a feature that has been fundamental in prevention science (Rohrbach 2014). Time spent in the field, learning with and from individuals in a target population, builds trust between researchers and participants, engendering a sense of obligation and accountability in researchers to serve the needs of the communities in which we work. Personal contact can have scientific value as well, leading to unexpected discoveries, new ideas, and personal rewards, which often fuel the best scientific work.

Researchers using remote interventions can take steps to avoid misunderstandings and mismatches and promote the human connections with participants that motivate beneficence. First, having a well-specified conceptual model articulating targets based on empirically identified needs in a population is essential (Fishbein et al. 2001). Online methods for recruiting and studying populations of interest can aid in shaping this conceptual model. When full-scale studies of population needs are not feasible, researchers can still use the Internet to collect preliminary data. Crowdsourcing services (e.g., Mechanical Turk, an online marketplace of distributed workers who respond to open calls to perform small tasks for pay) and commercial survey respondent pools (e.g., FluidSurvey) provide researchers with ready-made platforms to gather “quick and dirty” preliminary data through questionnaires and response harvesting (Mason and Suri 2012). Because these latter methods are a form of convenience sampling, researchers should be clear about sampling frames generated by these methods.

Second, direct experience and fieldwork with representative members of the target population during development can spark ideas for enhancing the benefit of an intervention (beneficence). The Play2Prevent Elm City Stories development process exemplifies the scientific and ethical value of extensive community-based development work (Hieftje et al. 2012):

Researchers at Yale University Play2Prevent Lab developed Elm City Stories, a video game designed to prevent risky teen behavior leading to HIV infection. To gain insights into the living environments, neighborhoods, and risky situations that their audience faced, they engaged teens in novel and creative activities such as “Photo feedback project” (teens taking and sharing pictures of their homes, neighborhoods, favorite hairstyles, peers, important adults), “Storytelling graphic illustration” (projective storytelling), and “My Life” (visual timeline of future aspirations and life goals). These activities directly informed the artwork, scenarios, and prevention strategies that appear in the resulting video game.

Although funding for such elaborate development is not always available, the principles of user-centered design (Abras et al. 2004) can guide the development process to whatever extent is feasible, assuring the needs, wants, and limitations of the target community are addressed at each stage. Usability testing involves direct observation of representative members interacting with an application or website (Brinck et al. 2001). During early design phases, in-person user testing under controlled conditions is recommended, especially those targeting high-risk populations. At all stages, direct observation, using remote video (e.g., Skype) when necessary, permits investigators to evaluate usability and see where the systems fail to meet users’ expectations.

Higher cost and longer timelines are generally the most significant barriers to staged development. The electronic marketplace moves quickly, and researchers developing interventions must balance accelerated development with adequate testing (Nilsen et al. 2012). One way to achieve this balance is to use a process that we proposed elsewhere, called “Continuous Evaluation of Evolving Behavioral Intervention Technologies” (Mohr et al. 2013a, b). Continuous Evaluation of Evolving Behavioral Intervention Technologies (CEEBIT) is a proposed methodology that continuously monitors use and clinical outcomes of multiple intervention technologies and could be used to test, prune, and refine different versions of the same intervention. This methodology allows researchers to monitor and evaluate the effectiveness of a range of applications and to eliminate the less efficacious. CEEBIT has the potential for improving the match and usefulness of an intervention by allowing step-wise validation and modification in small segments. However, for Behavioral Intervention Technologies to evolve and improve rapidly—and for researchers to study and understand participant responses to different iterations—IRBs will need to appreciate the function and value of iteration and avoid requiring researchers to “lock down” their intervention at the point of consent.

### Challenge No. 3: Integrating Interventions into Participants’ Lives While Minimizing Intrusiveness and Fatigue

Interventions that integrate seamlessly into the daily lives of research participants, especially through passive data collection and automatic tailoring, reduce risks of fatiguing participants and wasting time on unneeded elements. But, the risks of continuous monitoring and intervention are not yet known. As consent documents become increasingly streamlined as the NPRM envisions, it will be neither feasible nor desirable to inform participants of every possible risk in consent documents. Thus, researchers will need to be proactive about protecting participants from hidden risks.

Researchers from several different laboratories are developing human activity recognition systems to respond to problems as diverse as falls among the elderly, obesity, and smoking. Systems that use various combinations of accelerometers, gyroscopes, and depth video sensors (cameras that detect depth and 3D distance) are being tested to detect unsteadiness or actual falls and alert the older adult, family members, or caregivers. Dental implants (http://www.medicalnewstoday.com/articles/266402.php) and wearable jaw motion sensors and cameras (Fontana and Sazonov 2013; Sazonov et al. 2013) are being tested to recognize specific jaw movements, hand-to-mouth activity, and food intake for eventual use in promoting weight loss and smoking cessation.

To be most helpful, passive data collection would need to continuously monitor participants. But, the risks associated with continuous monitoring—even with full consent—are not well understood. As these examples illustrate, continuous monitoring and intervention alter the traditional meaning and commitment attached to being a research study participant. Always-on monitoring removes the boundary between research and the rest of life. Participating in such research is much different than the experience of accepting an interventionist into your home or receiving phone calls from staff administering measures. Both involve potential intrusions, but the experience is qualitatively different. Similarly, methods mentioned above, which monitor using smartphone microphones and speech recognition, could be perceived negatively by both participants and implementers as invading privacy in a “big brother is always watching” fashion. Plus, these devices capture data from other people in the environment who have not given consent. In other interventions, participants’ awareness of constant background monitoring of sound, video, location, or activity could make participants self-conscious or change their normal behavior in ways that are not yet well understood.

Tailoring interventions through requests for input or personalized “push” notifications, while potentially useful, also has the potential for harm. Researchers have documented the harmful effects of interruptions and information overload on productivity, memory, and emotions (Bailey and Konstan 2006). Interventions that increase human-machine interaction, requiring active responses to information and prompts, could thus introduce new stresses. For example, during the pre-intervention development stages of a text messaging intervention for teenagers (ARP), school staff and parents expressed concerns that ill-timed messages from the prevention program could cause interpersonal conflicts, such as texting prevention messages during a family meal. These anecdotal data led to our decision to avoid texting during school, dinner, and late night hours, even though teenagers stated that they preferred receiving late-night texts. Such trade-offs reflect the ethical tension between providing the most helpful and effective intervention and avoiding potential harm—trade-offs that are best explored and evaluated when researchers take the time to interact directly with stakeholders in developmental phases.

In the absence of clear data about the effect of continuous monitoring and of push notifications, researchers can take steps to reduce intrusion and burden and to support participant autonomy (respect for persons). First, researchers should provide a straightforward way for participants to temporarily pause data collection and participation. Second, researchers should be as transparent as possible about data collection and limit it to what is absolutely necessary. Third, because standards for privacy are fluid and culture bound, researchers testing always-on interventions should consult with members of the intended participant group and incorporate measures of fatigue and privacy invasion into all stages of development and field testing. Community advisory boards and other forms of direct input from community members can connect the researchers and IRBs to community standards, revealing values and expectations about privacy, intrusion, and safety. This helps align IRB concerns with participants’ foreseeable and reasonable expectations. For example, online behaviors indicate a willingness to exchange personal information for convenience, services, and personalization. There is a growing awareness that data we all contribute to linked systems provide benefits to society (Pentland 2013). Simultaneously, concerns about online privacy are growing, and a majority of individuals polled report feeling uncomfortable with a perceived loss of control over their personal information (Raine et al. 2013). In the social context of these countervailing and evolving tendencies, applications collecting movement or geolocation data might be familiar and acceptable to many and anathema to others. Thus, navigating these issues requires consultation with members of the intended participants’ group and should not be assumed known.

### Challenge No. 4: Setting Appropriate Expectations for Responding to Safety and Suicide Concerns

The principles of beneficence and respect for persons underscore the importance of ensuring the safety and well-being of research participants known to be vulnerable (e.g., suicide attempt history), as well as those who become so while participating (Fisher et al. 2002). We have a scientific and ethical responsibility to include people at risk for suicide and other risky behaviors in research to discover and test prevention opportunities and to assure scientific equity for these individuals (Pearson et al. 2001; Perrino et al. 2014). Yet, the scope of responsibility for monitoring and responding to safety risks when interventions are delivered over networked devices is still evolving, and care must be taken to set realistic expectations. The standards developed through small traditional clinical trials may not fit with large-scale online interventions.

Ginger.io is a private company that is developing algorithms to detect health-related patterns in smartphone sensor data. Ginger.io is currently investigating Mood Matters, a depression prevention program that uses activity and communication to alert individuals and their healthcare providers about fluctuations in depression and provides recommendations for responding. Individuals who use the program provide initial self-report information to train the program’s algorithms. The program collects and analyzes data behind the scenes and issues notifications when it determines that the user would benefit from taking action (such as contacting a friend or family member, exercising, or completing exercises assigned by a therapist or “coach”).

What responsibilities do researchers using Ginger.io have for detecting, evaluating, and actively responding to suicide-related material? One primary decision is establishing cut points in measures that would trigger real-time interventions. Where a known-vulnerable population is targeted, such adolescents with identified risk (e.g., suicide attempts), concerns and monitoring increase since the base rate and consequences of suicidal ideation and behavior are likely to be higher; however, researchers must also weigh the risks of too much direct monitoring: false positives, unwanted intrusions, and perceived invasion of privacy. Well-intentioned suicide prevention efforts by Facebook and its suicide prevention partners have been criticized along these lines. For example, a consumer watchdog agency publicized (PRNewswire 2015) an instance where a Facebook user was supposedly hospitalized inappropriately as a result of a friend using the “Report post” function that Facebook and its suicide prevention partners announced in 2015 (Facebook 2015). The veracity of the story is unconfirmed, but the media attention to this supposed incident reflects concerns about unintended negative consequences that could result from a social media company partnering with suicide prevention advocates to respond to suicide concerns.

If safety risks could be reliably detected, what responses are even possible? As with any human subjects decision, the answers will depend on the risks, benefits, and feasibility of options. For in-person interventions, safety protocols usually include standard responses (providing crisis intervention, telephone support, referrals to emergency, and mental health services). No such set of standard options yet exists for remote, asynchronous interactions. For example, in the USA, many states require in-state licensing to provide services, so intervening across state lines poses unique challenges. Furthermore, monitoring and responding to safety concerns might not be feasible for interventions capable of reaching very large participant groups. For example, in an ongoing randomized trial testing a universal suicide prevention program (Wyman et al. 2010) in 40 high schools, nearly 20,000 student participants in two different states completed online assessment of suicidal thoughts and behaviors in the past 12 months. Monitoring and intervening with high-risk individuals were not feasible. In this case, researchers provided information about accessing mental health resources to all participants within the online survey.

Another safety issue arises from technology failure rates. Websites or services participants rely on could experience outages. It is reasonable to expect that technologies developed on small research budgets will experience “bugs,” downtime, or even dramatic failures from time to time. When these occur, the consequences range from client annoyance and frustration to more serious failures in providing needed services. Mitigating frustrations is fairly straightforward—addressed by setting appropriate expectations of problems and technical support. For high-risk populations and newer interventions, the authors believe that researchers should budget enough to provide technical support and “customer assistance” for the duration of a research study and should specify support available after the study is completed. On the other hand, technology failures that hinder access to needed interventions or information usually require contingency planning. For example, one app currently in use provides mobile storage of a suicide safety plan and emergency contacts. Another analyzes glucose monitor results. Although the goals are quite different, failure of either of these apps to function could have serious negative consequences for the user. Researchers seeking to study the safety-planning app could provide recommendations and instructions for keeping a backup copy of plans and emergency contacts. Researchers studying new software for analysis of blood glucose could require patients to demonstrate that they have a secondary means of testing before enrolling them.

### Challenge No. 5: Safeguarding Newly Available Streams of Data

The NPRM states the following:

Society is in an information age. In all facets of one’s life information... is generated, stored, shared, analyzed, and often provides tremendous societal value. People share information about themselves with large numbers of people with the click of a button, and this trend of rapid and widespread sharing is only likely to grow. The increase in concern about unauthorized and inadvertent information disclosure, in combination with newer research techniques that increase the volume and nature of identifiable data suggest the need for the Common Rule to more explicitly address data security and privacy protection. (US Department of Health and Human Services 2015, §I.C.)

Public trust in prevention researchers depends on our ability to protect highly sensitive data from unintentional disclosure, and new regulations will require a greater degree of attention to security and privacy. Technology-heavy interventions and “big-data” explorations, especially those capturing or analyzing contextual information in the background, can generate sensitive data, both on the device and transmitted remotely to investigators. Sensor and geolocation data are especially sensitive and, in the wrong hands, could be abused or even lead to danger for the participant. Deductive disclosure (Sieber 2006) refers to the identification of participations based on triangulating combinations of reported or released information. Data that are geographically referenced, longitudinal, or multilevel (e.g., student, teacher, school, district or patient, clinic, community) are at higher risk for deductive disclosure. Adhering to reporting standards is one important way that researchers can guard against deductive disclosure (Inter-University Consortium for Political and Social Research 2012; Samarati and Sweeney 1998), but making use of evolving computational tools will help researchers address this concern more effectively.

New tools are available to protect participant confidentiality in single studies as well as in multi-study syntheses. These tools will be of great use to researchers as government requirements for data security are standardized and articulated, as the NPRM envisions. First, computational solutions can conceal individuals in a population. For example, we (CHB, CG) used a computer program to remove identifiers in a large dataset of text messages and log notes collected by a community partner (Wang et al., Automatic Classification of Communication Logs into Implementation Stages Via Text Analysis, unpublished). Embedded in the text were names and locations that could potentially have been used to identify individuals and link them to actions that they took, which was prohibited in the IRB agreement. Prior to analyzing these data, we “scrubbed” this dataset by automatically (a) sorting and enumerating every word; (b) identifying all names of persons, organizations, email addresses, and physical locations; (c) permuting these names randomly, and (d) replacing them with unique identifiers, such as PERSON1424 and LOCATION3449. We accomplished this process “in-house,” by data management personnel. In other words, the identified data is scrubbed before it leaves the agency that collected it. The table of names and tokens remains with the data collector. This and similar methods for automatic scrubbing allow rich analysis of the entire text including tokens, their relationship among each other, and the context in which they appear. This process de-identifies information, in a cost-efficient, fast manner, while maintaining accuracy and richness (Saygin et al. 2006). Scrubbing identifying information can also be done with audio and video recordings of participants. Computational approaches are under development to automatically detect and replace audio-visual information, such as a participant’s voice or face, with a fuzzy/blurred signal that still allows meaningful analysis (e.g., Bitouk et al. 2008; Chan et al. 2013; Gutta et al. 2005).

A second, more extensive method to protect identities is the use of a “hash function,” which is a way to provide an encrypted digital representation based on a combination of identifying information. An example of this is the globally unique identifier (GUID) used by the National Institute of Mental Health (NIMH) to allow linkages of individuals across different datasets in the National Database on Autism Research (Johnson et al. 2010). An algorithm is used to encrypt data drawn from each participant’s birth certificate, including full name, date, and place of birth. This yields a unique GUID that cannot be decrypted to recover the original information. Other studies that enter the same information would generate the same GUID, so individuals can be linked across different studies and analyzed without any indication of who the person is.

Third, methods of merging the same individuals across multiple datasets can be accomplished through the actions of a trusted broker (Boyd et al. 2009). As an intermediary, the broker links records across datasets based on relevant criteria (e.g., exclude all patients who “opt out” of using their medical record for research summaries), strips off identifiers or variables not permitted under an IRB-approved agreement, and makes the resulting data available to permitted researchers. We (PW, CHB) have used a trusted broker system to link longitudinal panel data from youth who are asked about their suicide ideation and behavior and social networks, thereby retaining anonymity.

One important limitation in planning data security protections is the lack of security of participants’ own devices. On the research institution side, disclosure by accident, hardware theft, or system intrusion can be mitigated by strong information security practices; however, the greatest threat to data security for participants is often the data stored on the participant’s own device. While traditional efficacy studies separate data collection from interventions, software-based interventions often place outcome data collection within the application itself, in part to reduce barriers to providing such data. Since mobile devices are often used in public venues or shared among family members or friends, interactions may be revealed inappropriately. Researchers cannot tell whose eyes are on the device at any given moment, opening the participant up to unintended exposure. Furthermore, many people do not secure their mobile devices from others in case of loss or theft (e.g., with strong passcodes and automatic or remote erasure). Researchers can require passcodes to open applications, but this may interfere with preferred interaction “styles,” creating barriers to effectiveness and adoption. Thus, researchers have little control over this threat to confidentiality other than by alerting participants and reminding them at critical junctures about data stored on the device. Such risks are not unique to mobile-mediated interventions—behavioral interventions have long used workbooks and journals that could be discovered—nevertheless, the concern may be heightened because mobile devices are more attractive to thieves, and digital information can be more easily exported and distributed (e.g., posted on a website) by a malicious person.

## Evolving Human Subjects Procedures to Match Current Needs: Progress Through Flexibility and Collaboration

Prevention science is in the midst of a technological revolution, and the USA is on the cusp of the first major update to federal human subjects policy since 1976 (Hudson and Collins 2015). This is an apt moment to consider the human subjects opportunities and challenges presented by technology in prevention research. Protecting human subjects aligns with the goals of prevention science—to maximize benefits and minimize risks to a population—good prevention science is inseparable from proper human subjects protection. As the Facebook informed-consent controversy illustrates (see above), researchers must be knowledgeable, sensitive, and proactive above and beyond what may or may not be required by IRB review. In that case, the Cornell IRB concluded that no review was required because their faculty member had access only to results, not to any individual, identifiable data (Cornell University Media Relations Office 2014). As partnerships with big-data companies increase and IRB oversight over lower-risk studies decreases (under proposed new rules of the NPRM), such situations requiring will become more common. Thus, prevention scientists, IRBs, commercial partners, and community members all have a vested interest in the integrity and flexibility of procedures designed to protect human subjects.

Table 1 provides a summary selection of goals, ethical tensions, and questions that prevention researchers, IRBs, and community members should consider together when planning a study involving technology. While the tensions are not fundamentally new, finding solutions in a new context is a critical challenge that we now face. Research on the impact of proposed solutions on comprehension, participation, and scientific productivity is needed. Prevention researchers can aid the development of an evidence base by reporting and examining their protocol decisions in empirical studies. Publicly posted consent documents, as proposed by the NPRM, could facilitate such research. Creative, realistic, and evidence-informed solutions could have benefits beyond studies that use technology by addressing concerns about IRB inflexibility and conservatism that predate the Internet. Thus, new technologies challenge us to update old assumptions and operating principles so that prevention science can continue to advance the well-being of research participants and their communities.

**Table 1:** Technology and human subjects protection: goals, ethical tensions, and protocol considerations

Human subjects goals	Ethical principles in tension	Considerations for human subjects protocols
Achieving adequate informed consent with procedures that are acceptable to participants in a digital age	Full comprehension and active consent (autonomy/respect for persons); matching participants’ expectations and preferences for computer-mediated interactions (respect for persons); scientific and social value of representative samples with diverse participants (justice, beneficence)	Have the researchers identified “critical” junctures where consent might be achieved for specific procedures or stages? Have researchers followed best practices for presenting online documentation? Have researchers considered the risk of participant dropout when planning online consent procedures?
Balancing opportunities for rapid development and broad reach with gaining adequate understanding of population needs	Preventive interventions accessible anywhere, anytime to anyone (justice, beneficence); avoiding and detecting harm in interventions deployed remotely asynchronously (respect for persons)	Do early stages of development include input and direct contact with intended population? Are there mechanisms for detecting cultural mismatches or other harms to sub-groups? Do research protocols allow flexibility for rapid iteration in response to feedback and discoveries?
Integrating data collection and intervention into participants’ lives while minimizing intrusiveness and fatigue	Reducing participant burden through passive data collection and personalization (respect for persons); increasing impact through frequent interaction (beneficence); protecting participants from stress of always-on monitoring or intervention (privacy, beneficence)	Can participants “pause” data collection or their participation without having to withdraw from the study? Have researchers provided adequate justification for all measures?
Setting appropriate expectations for responding to safety and suicide concerns	Protecting vulnerable/at-risk participants (beneficence, respect for persons); including at-risk individuals in research that could benefit them (justice); deploying interventions at massive scale to achieve broad impact for science and society (beneficence)	Have researchers communicated the timing and scope response that participants can expect in a crisis? What information and resources will participants be given in advance, and in the moment of a reported crisis? Can participants seek or be directed to help within the app/website/device?
Safeguarding newly available streams of data	Responsibility to take advantage of available data sources to promote public and individual health (beneficence); responsibility to protect confidentiality of research participants (confidentiality, respect for persons)	Have researchers explored technological solutions and trusted brokers for deidentifying sensitive data? Do researchers have reporting guidelines that reduce the risk of deductive disclosure? Have researchers considered and warned participants about the implications of a lost or stolen device?
